# ADXS11-001 immunotherapy targeting HPV-E7: updated survival and safety data from a phase 2 study in Indian women with recurrent/refractory cervical cancer

**DOI:** 10.1186/2051-1426-1-S1-P231

**Published:** 2013-11-07

**Authors:** Robert G Petit, Partha Basu

**Affiliations:** 1 Advaxis, Inc., Princeton, NJ, USA; 2 Chittaranjan National Cancer Institute, Kolkata, India

## 

ADXS11-001 immunotherapy is a live attenuated Listeria monocytogenes (*Lm*) bioengineered to secrete a HPV-16-E7 fusion protein targeting HPV transformed cells. The Lm vector serves as its own adjuvant and infects antigen presenting cells (APC) where it cross presents, stimulating MHC class I and II pathways resulting in specific T-cell immunity to tumors. Here we describe final 12 month overall survival data associated with ADXS11-001 administration in Lm-LLO-E7-015, a randomized P2 study conducted in India in 110 patients with recurrent cervical cancer; previously treated with chemotherapy, radiotherapy or both. Patients were randomized to either 1 cycle (3 doses) of ADXS11-001 at 1 x 10^9^ cfu or 4 doses of ADXS11-001 at 1 x 10^9^ cfu with cisplatin chemotherapy. Naprosyn and oral promethazine were given as premedications and a course of ampicillin was given 72 hours after infusion. Patients received CT scans at baseline and 3, 6, 9, 12 and 18 months. The primary endpoint is overall survival. As of May 17, 2013, the trial has completed enrollment and 110 patients received 264 doses of ADXS11-001. The percentage of patients at 12 months is 36% (39/110) and currently the 18 month survival is 22% (16/73). The response rate was 11% (6 CRs and 6 PR/110) with tumor responses observed in both treatment arms. 33 additional patients had stable disease > 3 months, for a disease control rate of 41% (45/110). Activity was observed against all high risk HPV strains detected. Two Grade 3 serious adverse events and 104 mild-moderate adverse events possibly related/related to ADXS11-001 treatment have been reported in 41% (45/110) of patients. The non-serious adverse events consisted predominately of transient, non-cumulative flu-like symptoms associated with infusion that either self-resolved or responded to symptomatic treatment. ADXS11-001 can be safely administered to patients with advanced cancer alone and in combination with chemotherapy. ADXS11-001 is well tolerated and presents a predictable and manageable safety profile. The addition of cisplatin to ADXS11-001 in this study did not significantly improve tumor responses or overall survival. Objective tumor responses included CR’s and apparent prolonged survival with minimal adverse experiences. Average duration of response in both treatment groups was 10.5 months. The 36% 12 month survival and 11% response rate observed in this recurrent disease setting is encouraging and suggests that ADXS11-001 is an active agent in recurrent cervical cancer. Final 18 month overall survival will be presented at the meeting.

